# Analysis of risk factors affecting union and refracture after combined surgery for congenital pseudarthrosis of the tibia: a retrospective study of 255 cases

**DOI:** 10.1186/s13023-022-02375-w

**Published:** 2022-06-23

**Authors:** Zhuoyang Li, Hui Yu, Yiyong Huang, Yaoxi Liu, Guanghui Zhu, Qian Tan, Haibo Mei, Ge Yang

**Affiliations:** 1 Department of Orthopedics, Hunan Children’s Hospital, Hunan, China; 2 Department of Orthopedics, College of Medicine, The First Affiliated Hospital, Zhejiang University, Hangzhou, China; 3 Department of Orthopedics, Guangdong Provincial People’s Hospital, Guangdong Academy of Medical Sciences, Guangdong, China

**Keywords:** Congenital pseudarthrosis of the tibia, Combined surgery, Union, Refracture

## Abstract

**Background:**

Congenital pseudarthrosis of the tibia (CPT) is a rare disease occurring in children. The aim of this study is to identify the factors affecting bone union and re-fracture after surgery for CPT and to provide reliable evidence for clinics.

**Methods:**

We collected the detailed information of 255 cases with Crawford IV CPT treated by combined surgery in our hospital from 2013 to 2020. Basic characteristics were recorded. Univariate variance and logistic regression analysis were used to compare the correlations between factors and outcomes.

**Results:**

92.5% of patients achieved primary union, 7.5% of patients had non-union and 13.3% of patients had re-fracture. Logistic regression analysis showed that age at index surgery (Coef. = 0.171, 95%CI 0.015–0.327, *P* = 0.032), and CPT location (Coef. = − 1.337, 95%CI − 2.218–0.456, *P* = 0.003) had statistical differences, while no factors had significant correlation with re-fracture. Furthermore, ROC curve showed that the optimal age threshold for first surgery was 2.37 years old.

**Conclusions:**

For patients with Crawford IV CPT treated by combined surgery, the younger the age at index surgery and the closer the CPT location to the distal end, the easier to achieve bone union.

## Background

Congenital pseudarthrosis of the tibia (CPT) is a rare condition occurring in 1:140,000 to 1:250,000 of live births [1]. Since Paget reported this disease in 1891, its etiology has not been clarified [2, 3]. It is usually manifested as developmental malformation of the tibia (anterolateral bowing), a narrow medullary cavity or fracture of the tibia with or without associated fibula fracture, and eventually, formation of tibial pseudarthrosis due to fibrous tissue between the fracture ends [4]. This can lead to malformation of the foot and ankle, abnormal growth and development of the remaining tibia, limb length discrepancy, and abnormal muscle strength in children [5]. Children without tibial fracture are usually treated with a brace to prevent the fracture. But when there is tibial fracture already, it is mainly treated by surgery. The objective of CPT treatment is to achieve long-term osseous healing of the pseudarthrosis to prevent limb length disparity and to further prevent re-fracture. The surgical approaches adopted by most surgeons include Ilizarov external fixation [6, 7], intramedullary rod combined with bone graft [8], and vascularized fibular graft (VFG) [9, 10], to achieve good mechanical stability. Other treatments include bone morphogenetic protein (BMP), and the use of bisphosphonates [11, 12] to shorten healing time by promoting osteogenesis. Currently, no single treatment is considered ideal [13]. Whatever treatment method is used, the goal is to achieve sustained bone healing after surgery.

Non-union and re-fracture are the main surgical complications of CPT, and patients often have to undergo re-operation, or these complication can even lead to disability and amputation [14]. Some literature has reported that the incidence of postoperative primary bone healing can reach 50–75% [15, 16]. However, after the bone is mature, only 40–60% can continue to maintain a good healing state. Once the fracture occurs again, the re-formed pseudarthrosis is difficult to heal itself, and the re-healing rate is only 35–50% [17, 18]. Therefore, in order to further improve the success rate of primary healing and reduce the incidence of re-fracture, it is very important to identify the factors affecting CPT bone union and re-fracture.

Previous literature has reported that bone union of CPT is the result of multiple factors, which may be related to the surgical method, age at index surgery, type I neurofibroma (NF-1), or congenital pseudarthrosis of the fibular (CPF) [19–21]. But there are no conclusion yet about which factors play a major role. Although there have been more and more studies on this topic in recent years, there are still few high-level evidence studies on its influencing factors. Therefore, the purpose of this retrospective study is to explore the related factors affecting the bone union and re-fracture after CPT surgery, in order to provide important evidence for the clinic.

## Method

### Basic information

Patients included in this study met the following criteria:

1. Patients with Crawford Type IV [22] CPT.
2. The same group of surgeons completed combined surgical treatment in our hospital [23, 24].
3. The follow-up was at least 2 years after the first surgery.
4. The data of patients were intact.

**Fig. 1:**
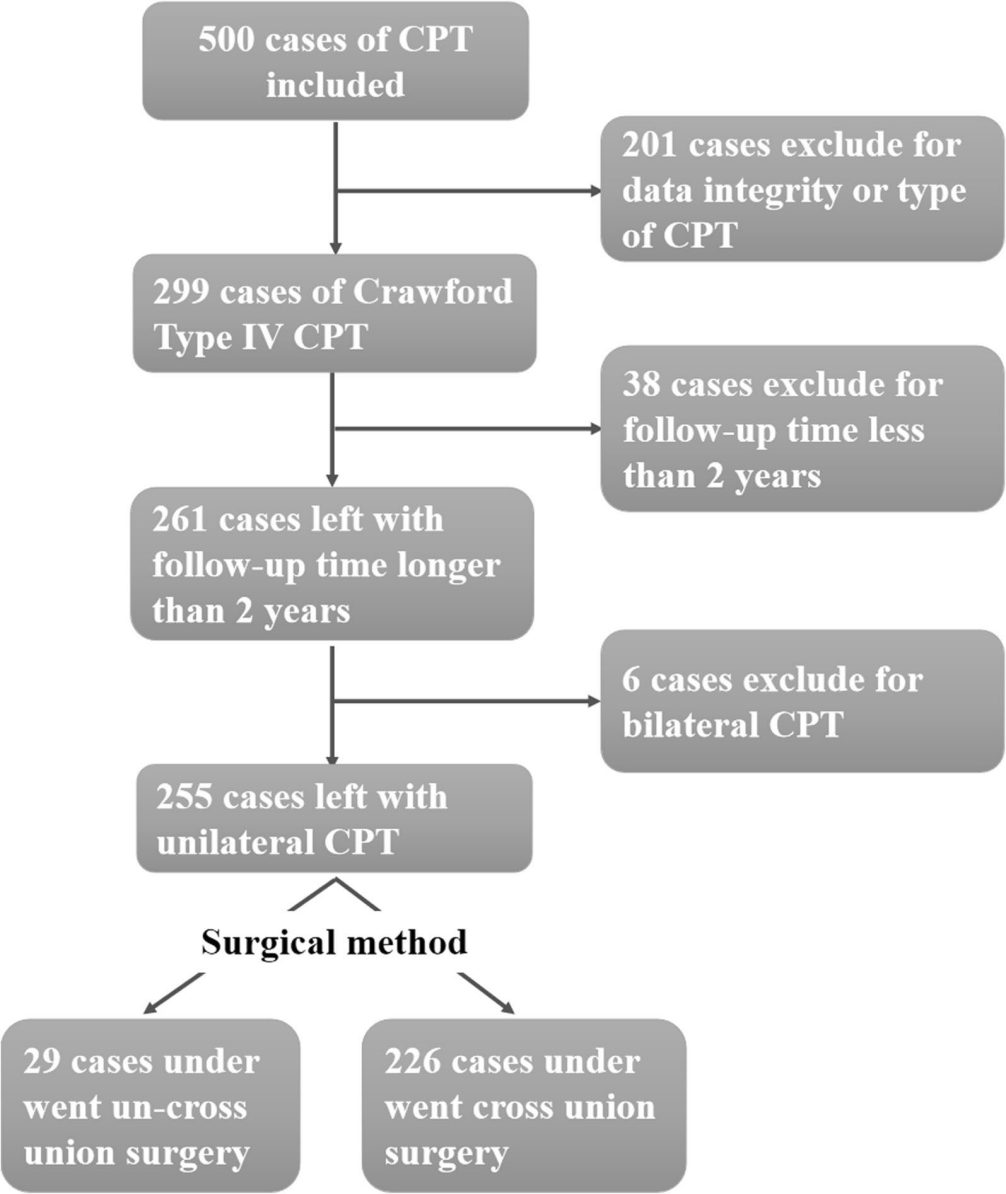
The inclusion strategy

The inclusion strategy is shown in Fig. 1, and 255 cases with Crawford IV CPT treated by combined surgery in our hospital from 2013 to 2020 were included. This study was approved by the ethics committee of our hospital, and informed consent was obtained from the patients’ parents.

We also collected the following influencing variables related to CPT bone union or re-fracture: gender, affected side, age at first fracture, age at index surgery, location of CPT in the tibia, whether combined with NF-1 (clinically diagnosed), fibula pseudarthrosis, etc. Moreover, the outcome variables were: (1) primary union of the tibia: union refers to the bone healing after the index surgery, and no additional surgical intervention is required, including delayed union which means healing takes longer than 3 months and less than 6 months, and eventually leads to bone union. Primary union is defined as a bridging callus appearing on at least three quarters of the cortex on anteroposterior and lateral X-ray films [25]; (2) refracture of the tibia: refracture refers to the reappearance of obvious fracture in the tibia on X-ray film after primary union.

The cases were divided into two groups according to the healing patterns after CPT surgery: primary union group and non-union group. In addition, we further divided the cases who had achieved primary union after the index surgery into two groups during the follow-up period: normal group and re-fracture group. The bone healing status and occurrence of re-fracture were determined by three surgeons independently. The diagnosis was only made when the opinions were consistent.

### Surgical procedures

All cases were treated with combined surgery, and the surgical procedures were based on the steps described in previous literature [23, 24]: 1. Excision of pathological tissue around tibial pseudarthrosis; 2. Fixation of the tibia through the ankle with an intramedullary rod; 3. Compression and fixation by Ilizarov external fixator; 4. Autogenous iliac wrapped bone transplantation.

In addition, according to the different methods of tibial fixation in the surgical procedure, we divided the surgical methods into two groups: 1, non-cross-union group: the tibia had been united alone; 2, cross-union group: both the tibia and fibula were united together. The cross-union procedure was based on previous literature [26, 27].

We improved the cross-union method slightly: if the patient had CPT alone, we used the "3-in-1" cross-union method to unite the affected tibia to the intact fibula; if the patient also had pseudarthrosis of the fibula, we used the "4-in-1" cross-union method to unite the 4 broken ends of the affected tibia and fibula together. All cases included in this study were performed by the same surgical group.

### Postoperative rehabilitation procedures

**Fig. 2:**
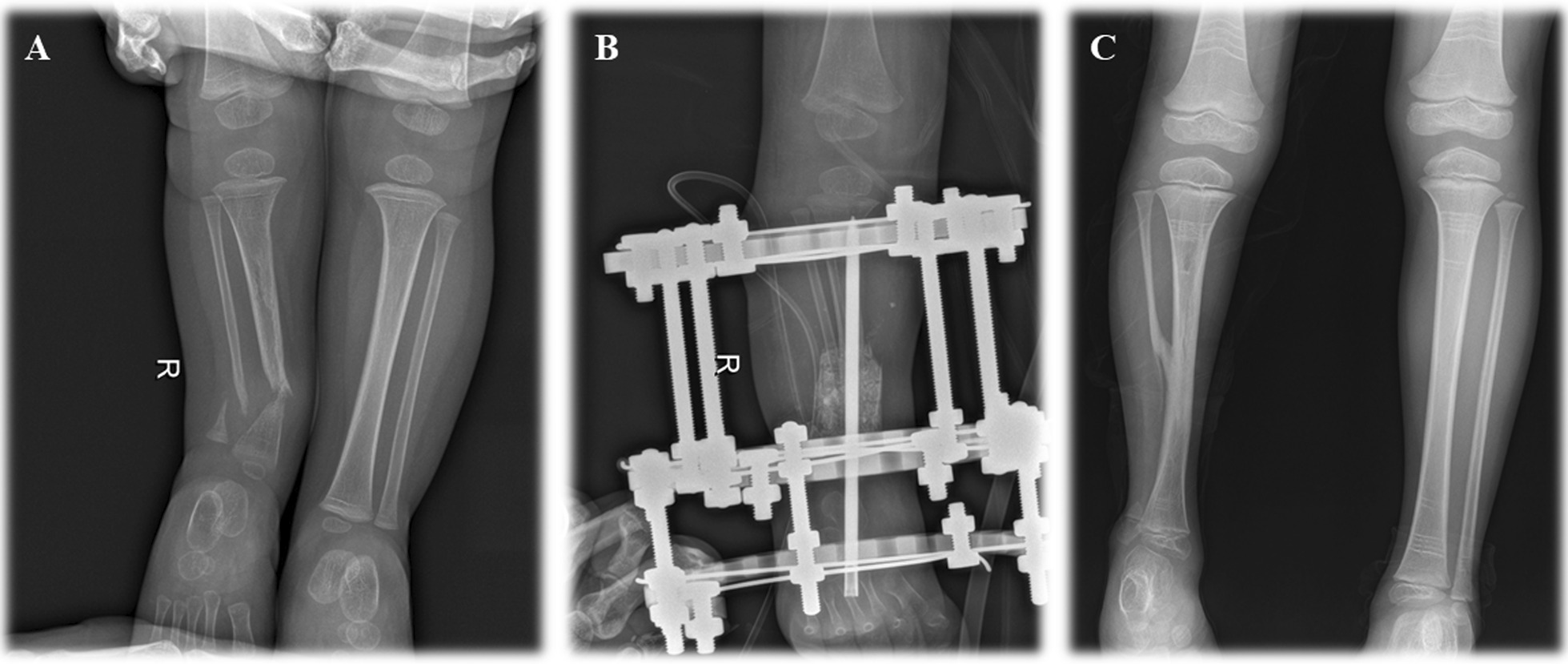
Preoperative and postoperative X-rays of combined surgery with cross-union. **A** preoperative X-ray; **B** postoperative X-ray within 7 days; **C** postoperative X-ray after primary union

**Fig. 3:**
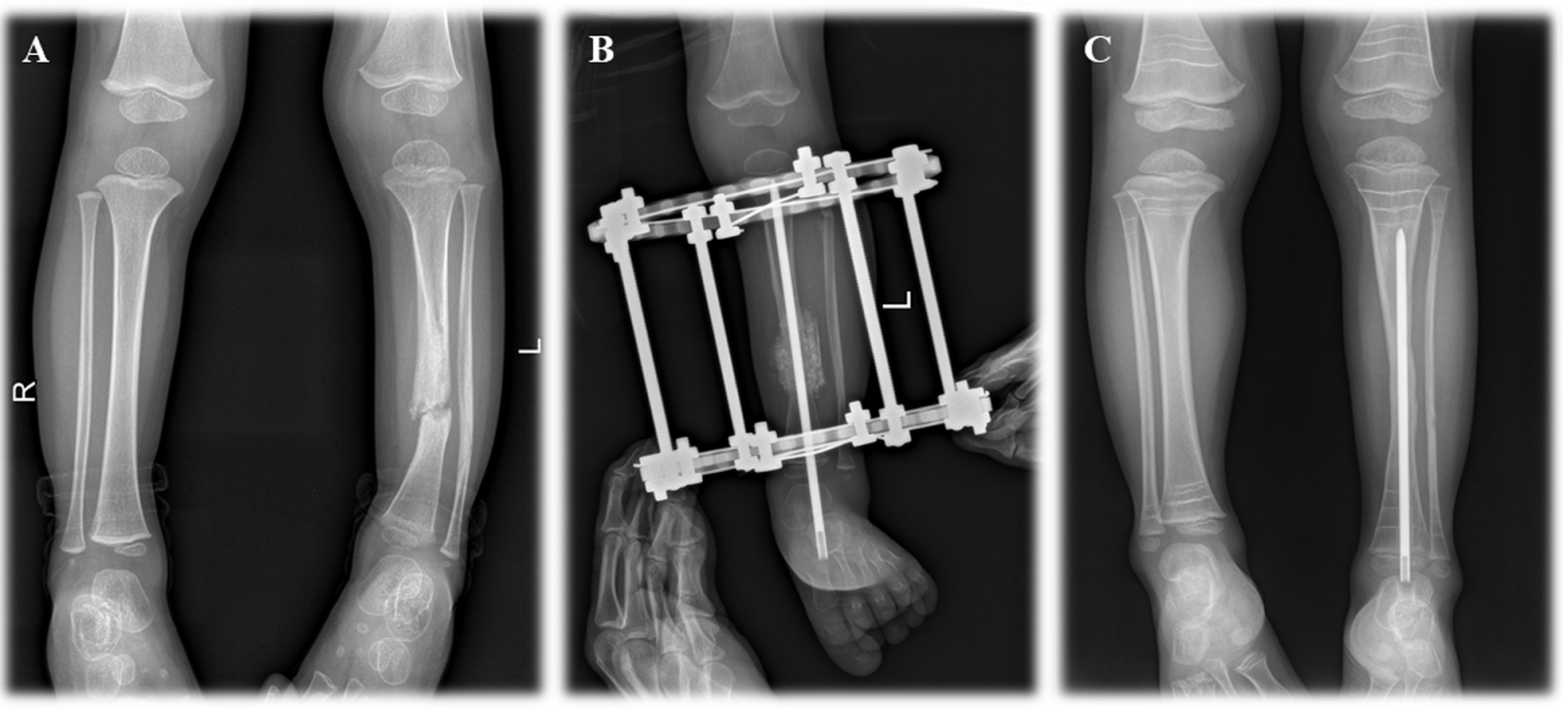
Preoperative and postoperative X-rays of combined surgery without cross-union. **A** preoperative X-ray; **B** postoperative X-ray within 7 days; **C** postoperative X-ray after primary union

Prophylactic antibiotics were used for 48 h after surgery. Positive and lateral X-ray films of lower limbs were taken 7 days after surgery and every 2 months after discharge. Pre- and post-operative X-rays of cross-union and non-cross-union are shown in Figs. 2 and 3.

When the radiographic union score for tibia (RUST) score [28] of CPT bone union reached 6 or above, the Ilizarov external fixator can be removed and fixed with long-leg tubular plaster for 2 months. After removing the plaster, the knee-ankle–foot brace should be used. At this time, the affected limb can walk with load. Patients should wear braces at all times, including when sleeping, except when taking a bath. When wearing braces, patients are allowed to participate in general sports.

### Statistical analysis

SPSS 23.0 software was used for statistical analysis in this study. Univariate variance analysis was used to compare differences in variables between groups. Bonferroni test was used as a post test. Meanwhile, univariate and multivariate logistic regression analyses were used to compare the correlations and odds ratios (OR) between variables and different event endpoints and calculate the corresponding 95% confidence intervals. Those variables with *P* < 0.1 in univariate analysis were counted as independent variables in multivariate regression analysis. Moreover, ROC curve was used to analyze the age threshold for optimal primary surgery. *P* < 0.05 was considered statistically significant.

## Results

### Basic information

A total of 255 cases were included in this study (see Table1 and Fig. 1 for details). There were 156 males and 99 females, 127 cases of left side, 128 cases of right side. The average follow-up time was 4.56 ± 1.71 years (2–8 years). By the time of case data collection, the average age for first fracture was 1.50 ± 1.71 years (0–11.1 years), 122 cases of younger than 1 years old, 92 cases of 1 to 3 years old, 41 cases of older than 3 years old. The average age for first surgery was 3.73 ± 3.13 years (0.1–16.7 years), 29 cases of younger than 1 year, 105 cases of 1 to 3 years old, and 121 cases of older than 3 years old. The average BMI was 16.50 ± 2.63 kg/m^2^. CPT was located in the proximal 1/3 of tibia in 6 cases, the middle 1/3 in 67 cases, and the distal 1/3 in 182 cases. There were 180 cases with combined NF-1 (73.7%), and 163 cases with fibula pseudarthrosis (63.9%).

**Table 1:** Basic characteristics of include cases

Variables	N/	Mean ± SD
Age at first Fracture		
< 1 y	122 (47.8%)	0.32 ± 0.29
1–3 y	92 (36.1%)	1.57 ± 0.49
≥ 3y	41 (16.1%)	4.87 ± 1.36
Age at index surgery		
< 1 y	29 (11.4%)	0.59 ± 0.24
1–3 y	105 (41.1%)	1.83 ± 0.50
≥ 3y	121 (47.5%)	6.14 ± 3.00
Follow-up time	4.56 ± 1.71 (2–8y)	
BMI	16.50 ± 2.63	
Side		
Left	127 (49.8%)	
Right	128 (50.2%)	
Gender		
Male	156 (61.2%)	
Female	99 (38.8%)	
Surgical method		
Non-cross union	29 (11.4%)	
Cross union	226 (88.6%)	
CPT location		
Proximal 1/3	6 (2.4%)	
Middle 1/3	67 (26.2%)	
Distal 1/3	182 (71.4%)	
Presence of NF-1		
Yes	180 (70.6%)	
No	75 (29.4%)	
Presence of CPF		
Yes	163 (63.9%)	
No	92 (36.1%)	
Refracture		
Yes	34 (13.3%)	
No	221 (86.7%)	
Nonunion		
Yes	19 (7.5%)	
No	236 (92.5%)	

*BMI* body mass index, *CPT* congenital pseudarthrosis of tibia, *NF-1* neurofibromatosis1, *CPF* congenital pseudarthrosis of fibula

According to the surgical methods, we divided the cases into 2 groups: non-cross-union and cross-union group. Basic information and outcomes of two groups were noted and analyzed (See Table 2 for details). There were 226 cases (88.6%) undergoing combined surgery with cross-union, while 29 cases (11.4%) were without cross-union. The results showed that there was no significant difference in basic information between the two groups (*P* > 0.05).

**Table 2:** Variance analysis of different surgical methods

Variables	Non-Cross union	Cross union	*P*
*Surgical methods*			
Age at index surgery	3.55 ± 2.62	3.71 ± 3.17	0.802
Age at first fracture	1.09 ± 1.30	1.56 ± 1.75	0.182
BMI	17.04 ± 3.34	16.26 ± 2.40	0.103
Presence of NF-1			
Yes	19(65.5%)	161 (71.2%)	0.845
No	10 (34.5%)	65 (28.8%)	
Presence of CPF			
Yes	18 (62.1%)	147 (65.0%)	0.973
No	11 (37.9%)	79 (35.0%)	
Refracture			
Yes	3 (10.3%)	30 (13.3%)	0.805
No	26 (89.7%)	196 (86.7%)	
Nonunion			
Yes	1 (3.4%)	18 (8.0%)	0.453
No	28 (96.6%)	208 (92.0%)	
CPT Location			
Proximal 1/3	2(6.9%)	4 (1.8%)	0.054
Middle 1/3	9 (31.0%)	58 (25.7%)	
Distal 1/3	18 (62.1%)	164 (72.6%)	

*BMI* body mass index, *NF-1* neurofibromatosis1, *CPF* congenital pseudarthrosis of fibula, *CPT* congenital pseudarthrosis of tibia

Moreover, 19 cases had bone non-union (7.5%), and 34 cases had re-fracture after bone union (13.3%). Separately, there was 1 case of non-union (3.4%) and 3 cases of refracture (10.3%) in the non-cross-union group, and 18 cases of non-union (8%) and 30 cases of refracture (13.3%) in the cross-union group. Variance analysis of outcomes also showed no significant difference (*P* = 0.453, 0.805).

### Bone union status after CPT surgery

Of the 255 cases, 236 cases (92.5%) had primary bone union after CPT surgery, while 19 cases (7.5%) had non-union (See Table 3 for details).

**Table 3:** Related Variables of nonunion

Variables	Yes	No	*P*
*Nonunion*			
**Age at index surgery**	**5.61 ± 3.96**	**3.58 ± 3.01**	**0.006**
**Age at first fracture**	**2.26 ± 2.01**	**1.44 ± 1.67**	**0.044**
BMI	16.25 ± 2.25	16.52 ± 2.66	0.671
Gender			
Male	11 (57.9%)	141 (59.7%)	0.875
Female	8 (42.1%)	95 (40.3%)	
Presence of NF-1			
Yes	15 (78.9%)	165 (69.9%)	0.408
No	4 (21.1%)	71 (30.1%)	
Presence of CPF			
Yes	14 (73.7%)	152 (64.4%)	0.416
No	5 (26.3%)	84 (35.6%)	
**CPT Location**			
** Proximal 1/3**	**3 (15.8%)**	**3 (1.3%)**	**0.004**
** Middle 1/3**	**6 (31.6%)**	**61 (25.8%)**	
** Distal 1/3**	**10 (52.6%)**	**172 (72.9%)**	
Surgical methods			
Non-cross union	1 (5.3%)	28 (11.9%)	0.385
Cross union	18 (94.7%)	208 (88.1%)	

The [bold] means* p* < 0.05. There are significant differences

*BMI* body mass index, *NF-1* neurofibromatosis1, *CPF* congenital pseudarthrosis of fibula, *CPT* congenital pseudarthrosis of tibia

Statistical analysis of bone union, and non-union groups showed that there were significant statistical differences in age at first fracture (*P* = 0.044), age at index surgery (*P* = 0.006) and CPT location (*P* = 0.004). The ages at first fracture and index surgery in patients with bone union were less than those of the patients with non-union, and the closer the location was to the proximal end, poor healing was more likely to occur (72.9% and 52.6%).

**Fig. 4:**
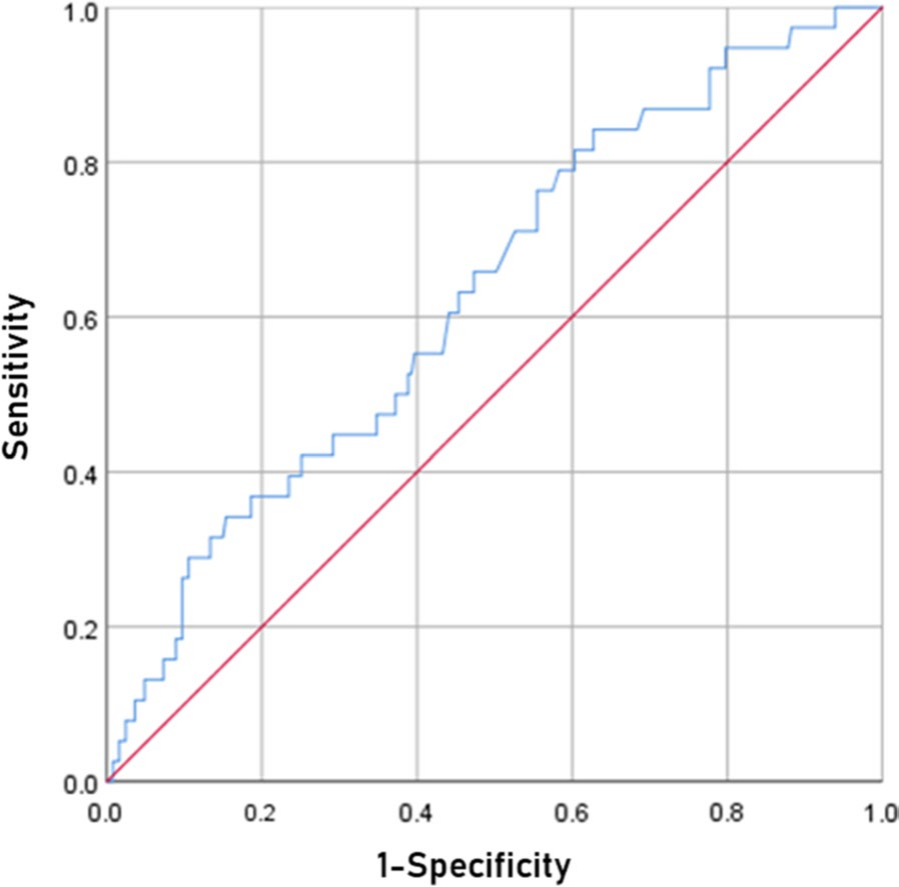
This figure shows the ROC Curve. The abscissa is 1-specificity, the ordinate is sensitivity. According to the curve, cut-off value is 2.37 years old

Logistic regression analyses were conducted for relevance between variables and CPT bone union (see Table 4). The results showed that two variables had statistical differences: age at index surgery (coef. = 0.171, 95%CI 0.015–0.327, *P* = 0.032) and CPT location (coef. =  − 1.337, 95%CI − 2.218 − 0.456, *P* = 0.003). Furthermore, ROC curve was used to calculate the optimal age threshold for first surgery of bone union, and the results showed that ROC = 2.37 years old (see Fig. 4).

**Table 4:** Logistic regression analysis of Nonunion and other variables included

Variables	Coef	95% CI	*P*
*Nonunion*			
**Age at index surgery**	**0.171**	**0.015–0.327**	**0.032**
Age at first fracture	0.057	− 0.231–0.345	0.699
BMI	0.013	− 0.190–0.216	0.900
Presence of NF-1	0.600	− 0.672–1.872	0.355
Presence of CPF	0.901	− 0.313–2.115	0.146
**CPT location**	− **1.337**	− **2.218–0.456**	**0.003**
Surgical methods	1.222	− 1.026–3.470	0.287

The [bold] means* p* < 0.05. There are significant differences

*BMI* body mass index, *NF-1* neurofibromatosis1, *CPF* congenital pseudarthrosis of fibula, *CPT* congenital pseudarthrosis of tibia

### Re-fracture after CPT surgery

Of the 255 cases, 34 cases (13.3%) had re-fracture after surgery (see Table 5 for details). Interestingly, we evaluated and analyzed the basic information and the outcomes of the normal and re-fracture groups. The results showed that no matter in univariate variance analysis or logistic regression analysis, there was no factor significantly associated with refracture (see Tables 5, 6).

**Table 5:** Related Variables of refracture

Variables	Yes	No	*P*
*Refracture*			
Age at index surgery	3.75 ± 2.31	3.73 ± 3.24	0.966
Age at first fracture	1.56 ± 1.78	1.49 ± 1.70	0.829
BMI	16.47 ± 2.18	16.50 ± 2.69	0.955
Gender			
Male	21 (61.8%)	135 (61.1%)	0.940
Female	13 (38.2%)	86 (38.9%)	
Presence of NF-1			
Yes	26 (76.5%)	154 (69.7%)	0.421
No	8 (23.5%)	67 (30.3%)	
Presence of CPF			
Yes	22 (64.7%)	144 (65.2%)	0.959
No	12 (35.3%)	77 (34.8%)	
CPT location			
Proximal 1/3	1(2.9%)	5 (2.3%)	0.108
Middle 1/3	13 (38.2%)	54 (24.4%)	
Distal 1/3	20 (58.8%)	162 (73.3%)	
Surgical methods			
Non-cross union	4 (11.8%)	25 (11.3%)	0.939
Cross union	30 (88.2%)	196 (88.7%)	

*BMI* body mass index, *NF-1* neurofibromatosis1, *CPF* congenital pseudarthrosis of fibula, *CPT* congenital pseudarthrosis of tibia

## Discussion

CPT is a difficult problem in pediatric orthopedics because of the unclear pathogenesis, difficulties in treatment and long treatment time since being reported. After years of clinical practice, it has been proved that combined surgery can make up for the shortcomings of single surgery, and greatly improve the probability of primary bone union. Combined surgery is the most common treatment at present [15]. In this study, the rate of primary bone union in patients with CPT reached up to 92.5%, which is similar to the results of previous studies. Dobbs et al. reported that the rate of primary bone union in CPT patients treated with intramedullary rods could reach up to 85.7% [16]. Shah et al. counted 119 cases from multiple centers with different surgical methods, and the overall bone-healing rate was 86% [17]. Although the results of primary bone union rate are encouraging, it has been pointed out that as the patient’s bones mature, the good and stable primary healing bone may continue to deteriorate and evolve into re-fracture [4]. Our results showed that 80.2% cases with primary union still maintained good status. This is better than previously reported using the single surgical technique [5, 29, 30]. From this point, we find that combined surgery plays an important role in improving the primary bone union rate and reducing the re-fracture rate. Our previous studies have also proved this conclusion. Ilizarov fixation provides a high union rate and good end contact. Intramedullary rod fixation stabilizes the pseudarthrosis and avoids re-fracture. Autologous bone transplantation makes the graft closely contact with the pseudarthrosis over a large area to enhance mechanical stability and provide a unique biological environment to promote union [23].

The procedure of the cross-union method was first used by Johnston [31]. He emphasized the effect of fibula union for bone union and refracture. Subsequently, Choi et al. formally proposed the concept of cross-union and reported cases with cross-union in 2011 [26]. They united the two ends of the fibula and tibia with iliac bone graft which was named “4-in-1 osteosynthesis”. They also reported that the bone healing rate of 8 cases was 100% without a refracture, with an average follow-up of 7.4 years. Moreover, in a very recent meta-analysis of various surgical methods for the treatment of CPT, the results showed that cross-union achieved the best clinical results in both union rate (100%) and re-fracture rate (22.5%), which may be related to the increase of bone contact cross-sectional area [32]. Although the sample size of these reports was not very large, the results of 100% bone union were encouraging, which showed the importance of cross-union for CPT treatment. Therefore, more and more surgeons believe that cross-union is the gold standard for CPT treatment.

**Table 6:** Logistic regression analysis of refracture and other variables included

Variables	Coef	95% CI	*P*
*Refracture*			
Age at index surgery	0.002	− 0.146–0.149	0.983
Age at first fracture	0.035	− 0.227–0.296	0.795
BMI	− 0.050	− 0.224–0.124	0.572
Presence of NF-1	0.404	− 0.513–1.321	0.387
Presence of CPF	0.287	− 0.534–1.109	0.493
CPT Location	− 0.577	− 1.261–0.107	0.098
Surgical methods	0.219	− 1.110–1.549	0.747

*BMI* body mass index, *NF-1* neurofibromatosis1, *CPF* congenital pseudarthrosis of fibula, *CPT* congenital pseudarthrosis of tibia

We also realized the importance of the cross-union technique, and modified “4-in-1 osteosynthesis” in a previous study [33]. For patients with intact fibula, “3-in-1 osteosynthesis” was used to increase the cross-sectional area of the segment. 17 patients were followed up for 4 years without further refracture (a success rate of 100%). The results showed that “3-in-1” is an effective surgical method for the treatment of type A CPT with intact fibula. In this study, we expanded the number of cross-union cases. The results showed that after an average follow-up of 4.6 years the bone union rate of 226 cross-union cases was 88.6%, 29 cases (11.4%) failed to achieve bone union and the re-fracture rate was 13.3%. The success rate of treatment was not inferior to those reported in other literature. The small decrease in union rate might be related to a variety of factors, such as an increase in sample size, the amount of complex surgery, and the incidence of complications. But there is no doubt that combined surgery with cross-union is an extremely effective surgical method for CPT treatment. Interestingly, different from previous literature, there was no significant difference in outcomes between the cross-union and non-cross-union groups. Since the two groups were different in size the statistical analysis of this may not be at a sufficient power to state that this lack of difference is real.

The correlation between age of treatment and CPT prognosis is controversial. Some studies report the incidence rate of postoperative non-union as being higher in children younger than 3.Boero et al. [34] found that children with CPT should avoid surgery before the age of 3 and try to delay the surgery to the age of 5. Kristiansen et al. [35] held that the younger the age at surgery, the higher the risk of tibial alignment abnormalities and re-fracture. Cho et al. [19] followed up 23 children for 9 years and found that re-fracture is more likely to occur in children younger than 4. However, in recent years, more and more literature has indicated that surgery is safe and effective in younger children (younger than 3). And early accurate surgical treatment can effectively promote the normal development of the diseased limb and reduce the length difference. Joseph et al. [36] reported that the bone union rate of children younger than 3 years old could reach 92.3% after treatment. Tan et al. [10] followed up 11 cases of children with fibular vascular pedicle transplantation for 11 years and found no significant correlation between surgical age and postoperative re-fracture. In this study, the age of primary surgery in the bone union group was significantly younger than that in the non-union groups. ROC curve was used to calculate the optimal age threshold for surgery, which was 2.37 years old. This confirms the results of previous literature that early surgical intervention in patients aged 1 to 3 is safe and feasible. The reason for the contradictory results may be related to the surgical method applied. Most surgical methods in previous literature were not fixed, and the samples were generally small, which can lead to selection bias.

Few studies have reported on the relationship between CPT location and postoperative bone union or re-fracture. It is generally believed that CPT located in the distal 1/3 of the tibia is the most common, while that in the middle and upper 1/3 is rare. Some studies have reported that the incidence rate of proximal CPT is only 2%, and 29% of CPT location will change over time, making it difficult to diagnose the lesion location [1]. Nguyen et al. [37] reported that 29 patients with CPT were all located in the distal 1/3. However, the samples of previous literature were too small to completely describe the CPT location accurately. In this study, of the 255 cases, location in the distal 1/3 accounted for 71.4%, but location in the proximal 1/3 accounted for only 2.4%, which is similar to the results of previous literature. Meanwhile, our analysis found that CPT location is significantly correlated with postoperative bone union: the closer the CPT location is to the proximal end, the greater the possibility of postoperative poor bone healing (*P* = 0.003). Therefore, we suggest that patients with proximal CPT should be treated with a more aggressive and comprehensive approach to reduce the risk of postoperative poor bone healing, thereby reducing the incidence of re-fracture. The samples of proximal CPT were few, so there may be sample bias, which requires further studies with more samples to confirm this conclusion. Moreover, this study found that gender, BMI and combined NF-1 or CPF were not significantly correlated with poor bone healing and re-fracture after CPT surgery. This is also similar to the results of previous studies [38].

This study has some limitations. Although the total sample in this study is much bigger than that of previous literature, the independent sample size of each sub-group still does not reach the ideal state, which may lead to potential bias. Secondly, this study only includes children with Crawford IV CPT and combined surgery was used for treatment only, which are both advantages and possible disadvantages of this study. We could not completely avoid selection bias, which may have affected the results. This study was based on retrospective analysis, and was therefore unable to control some variables, such as treatment compliance, daily activity, economic status, etc., which may all have affected the prognosis of surgery. In addition, the follow-up time in this study was not long enough to determine the actual incidence of re-fracture, which has some impact on our analysis of risk factors for re-fracture. All the results came from the same surgical team at the same center. Determining whether the results of this study are applicable to the majority of children with CPT requires more studies with more samples in more centers.

## Conclusion

In conclusion, for 255 cases with Crawford IV CPT treated by combined surgery the union rate was 92.5%, the refracture rate was 13.3% and the rate of union without refracture was 80.2%. Moreover, postoperative bone union is related to age at index surgery and CPT location. The younger the age at index surgery, and the closer the CPT location to the distal end, the easier to achieve bone union.

## Data Availability

Data are available by request.

## Abbreviations

CPT: Congenital pseudarthrosis of tibia
VFG: Vascularized fibular graft
BMP: Bone morphogenic protein
NF-1: Type I neurofibromatosis
CPF: Congenital pseudarthrosis of fibula
